# Heme Oxygenase Induction Suppresses Hepatic Hepcidin and Rescues Ferroportin and Ferritin Expression in Obese Mice

**DOI:** 10.1155/2017/4964571

**Published:** 2017-09-14

**Authors:** Nitin Puri, Yevgeniy Arefiev, Robert Chao, David Sacerdoti, Hibba Chaudry, Alexandra Nichols, Krithika Srikanthan, Athar Nawab, Dana Sharma, Vishal Hari Lakhani, Rebecca Klug, Komal Sodhi, Stephen J. Peterson

**Affiliations:** ^1^Department of Physiology & Pharmacology, University of Toledo College of Medicine, Toledo, OH 43614, USA; ^2^Department of Medicine, Weill Cornell Medicine/NYP Brooklyn Methodist Hospital, Brooklyn, NY 11215, USA; ^3^Department of Clinical and Experimental Medicine, University of Padova, Padoua, Italy; ^4^Departments of Medicine and Physiology, Marshall University School of Medicine, Huntington, WV, USA

## Abstract

Hepcidin, a phase II reactant secreted by hepatocytes, regulates cellular iron levels by increasing internalization of ferroportin-a transmembrane protein facilitating egress of cellular iron. Chronic low-grade inflammatory states, such as obesity, have been shown to increase oxidative stress and enhance hepcidin secretion from hepatocytes and macrophages. Heme-heme oxygenase (HO) is a stress response system which reduces oxidative stress. We investigated the effects of HO-1 induction on hepatic hepcidin levels and on iron homeostasis in hepatic tissues from lean and obese mice. Obese mice exhibited hyperglycemia (*p* < 0.05); increased levels of proinflammatory cytokines (MCP-1, IL-6, *p* < 0.05); oxidative stress (*p* < 0.05); and increased hepatic hepcidin levels (*p* < 0.05). Enhancement of hepcidin was reflected in the reduced expression of ferroportin in obese mice (*p* < 0.05). However, this effect is accompanied by a significant decline in ferritin expression. Additionally, there are reduced insulin receptor phosphorylation and attenuation of metabolic regulators pAMPK, pAKT, and pLKB1. Cobalt protoporphyrin- (CoPP-) induced HO-1 upregulation in obese mice reversed these alterations (*p* < 0.05), while attenuating hepatic hepcidin levels. These effects of CoPP were prevented in obese mice concurrently exposed to an inhibitor of HO (SnMP) (*p* < 0.05). Our results highlight a modulatory effect of HO on iron homeostasis mediated through the suppression of hepatic hepcidin.

## 1. Introduction

Oxidative stress contributes to the development, and/or progression, of numerous acute and chronic pathological states. Contextually, the role of reactive oxygen species (ROS) in complex multifactorial disorders, such as obesity, fatty liver, and metabolic syndrome has received widespread attention, and obesity is now characterized as a chronic low-grade inflammatory state. In this regard, intracellular free iron is cytotoxic, drives the Fenton reaction, and exacerbates oxidative stress [1–4]. Intracellular iron homeostasis is regulated by a complex crosstalk between the peptide-hepcidin, a near ubiquitous transmembrane protein ferroportin, and an intracellular iron binding protein, ferritin. Hepcidin downregulates ferroportin and reduces egress of excess intracellular irons that can then bind to IRP-1 and induce iron responsive genes, including ferritin [5–7]. In the face of an insufficient ferritin upregulation, free iron can exert its prooxidant and cytotoxic effects. These molecular events feed-forward on ROS and contribute to the escalation of pathophysiological alterations in conditions, such as NAFLD and obesity [1–3, 8, 9]. The crucial importance of ferritin in the protection of the liver to oxidative insult is further corroborated in ischemia reperfusion injury [10].

Upregulation of the cytoprotective enzyme system, heme oxygenase (HO), is an essential process and has been shown in the past to alleviate and prevent several pathological conditions, including adipocyte dysfunction and obesity [8, 11–17]. Heme-HO system metabolizes heme to equimolar concentrations of biliverdin (BV), carbon monoxide (CO), and iron. While biliverdin is a potent natural antioxidant, alleviating lipid peroxidation, CO has vasodilatory and antiapoptotic properties [4]. Pharmacogenetic induction of the inducible HO isoform (HO-1) leads to attenuation of adiposity along with restoration of insulin sensitivity and abatement of chronic inflammation, cardiomyopathy [18], and pathophysiological insults frequently associated with diet induced obesity [2, 3, 8, 11, 19–22]. These effects of HO-1 induction have been associated with its stimulatory effects on adiponectin secretion, with a subsequent activation of pLKB1/pAMPK-dependent pathways [23].

Serum hepcidin and hepatic iron content are positively correlated with obesity in human subjects [24], an observation corroborated in obese adolescents and children [25]. For the current study, we hypothesize that HO-1 induction will restore hepatic redox balance, reduce hepcidin secretion, and restore mitochondrial integrity. Observations were first contrasted in experiments conducted in lean and obese mice followed by characterization of a modulatory role of HO-1 by conducting experiments in obese mice treated with and without CoPP, in the absence or presence of an HO-1 inhibitor, SnMP. Our results show that obese mice have increased hepatic levels of hepcidin complemented by lower expression of ferroportin and ferritin with increased levels of NRF2 and gp91phox, an indication of oxidative stress. CoPP-mediated HO-1 induction reverses these alterations in obese mice along with a significant improvement in insulin sensitivity, decreased hepcidin, and increased ferroportin and mitochondrial Mfn-1/2 levels. The fact that these rescue effects of CoPP in obese mice are prevented in mice cotreated with SnMP corroborates the regulatory role of heme-HO system in iron homeostasis.

## 2. Materials and Methods

### 2.1. Animal Treatment

All experiments were performed following an IACUC approved protocol in accordance with the* NIH Guide for the Care and Use of Laboratory Animals*. Male obese (ob) mice (B6v-Lep ob/J) were purchased from Harlan (Chicago, IL) at the age of 7 weeks and used at the age of 8 weeks. Age- and sex-matched lean mice (B6.V, lean; Harlan) were used as controls. Mice were fed a normal chow diet and had free access to water. Body weights of the mice at the beginning of the treatment were 23.7 ± 2.5 g for lean and 35.7 ± 4.7 g for obese mice. Glucose levels were 129.2 ± 10.8 mg/dl and 209.7 ± 6.8 mg/dl for lean and obese mice, respectively.

Glucose monitoring was performed using an automated analyzer (Life scan, Milpitas, CA). Cobalt protoporphyrin (CoPP), an inducer of HO-1, was given intraperitoneally once a week (3 mg/kg) for 6 weeks to obese mice. CoPP plus stannous mesoporphyrin (SnMP), to inhibit HO activity, was administered intraperitoneally three times a week (20 mg/kg) for 6 weeks [26] to ascertain that any effects of CoPP treatment were related to increased HO activity. Metalloporphyrins were dissolved in 10 mmol/l Tris base, and the pH was adjusted to pH 7.8 with 0.1 N HCl. A Tris/HCl solution free of Metalloporphyrins was used to inject control animals. The animals were equally divided into four groups: (1) lean, (2) obese (3) obese CoPP, and (4) obese CoPPSnMP. Food intake did not change in the mice treated with the various treatments. At the time of sacrifice, the body weight of all mice was measured. After a 6-hour fast, mice were anesthetized with sodium pentobarbital (65 mg/kg, i.p.) and blood was obtained from a tail vein for glucose measurement using a glucometer. Blood samples were collected in K_3_EDTA tubes at sacrifice and the plasma was separated. Samples were flash frozen in liquid nitrogen and maintained at −80°C until needed.

### 2.2. Plasma Cytokines Measurements

TNF-alpha and IL-6 in plasma were determined using an ELISA assay (Pierce Biotechnology, Inc., Woburn, MA) as previously described [27].

### 2.3. Western Blot Analysis

#### 2.3.1. Tissue Preparation for Western Blot

After 6 weeks of treatments the mice were killed, and the liver was harvested, drained of blood, and flash frozen in liquid nitrogen. Livers were maintained at −80°C until needed.

#### 2.3.2. Fractionation of Microsomal and Nuclear Fractions

Frozen pieces of liver were placed in a homogenizing buffer consisting in mmol/l of 10 phosphate buffer, 250 sucrose, 1 EDTA, 0.1 PMSF, and 0.1% v/v Tergitol, pH 7.5. The nuclear fractions were obtained using Nuclear Extraction Reagents (Thermo Scientific, Rockford, IL, USA) according to the manufacturer's protocol. Homogenates were centrifuged at 27.000*g* for 10 min at 4°C to remove cell debris; then the supernatant was isolated and protein levels were assayed (Bradford method). After mixing samples with loading buffer (Tris-Cl 50 mM, SDS 10% w/v, glycerol 10% v/v, 2-mercaptoethanol 10% v/v, and bromophenol blue 0.04%) at a of ratio 4 : 1, they were boiled for 5 min. In brief, 20 *μ*g protein was loaded onto 8% or 12% SDS-polyacrylamide gels and subjected to electrophoresis (90 V, 100 min). The separated proteins were transferred onto 0.45 *μ*m PVDF membranes (Millipore, Billerica, MA, USA) for 1 h (140 mA). After transfer, the blots were incubated with Blocking Buffer Solution (Odyssey, LI-COR Biosciences, Lincoln, NE, USA) at room temperature for 1 hour with constant shaking. Then, the membranes were incubated with various antibodies against specific proteins: HO-1, HO-2, and ferritin heavy chain (Cell Signaling Technology, Boston, MA, USA), ferroportin-1 (Santa Cruz Biotechnology, CA, USNFR-2 (Abcam), gp91 phox, UCP1, and UCP2 (Abcam), insulin receptor beta Cell Signaling Technology), insulin receptor phosphorylated at Tyr 1146 (Cell Signaling Technology) and at Tyr 1322 (Assay Design, Ann Arbor, MI, USA), and AKT, pAKT, AMPK, pAMPK, Mfn1, and Mfn 2 (Cell Signaling Technology) at 4°C overnight with constant shaking. The blots were washed in TBS 1x and subsequently were incubated with fluorophore-conjugated secondary antibodies (IRDye, LI-COR Biosciences). Finally, the blots were developed using an Infrared Imaging System (Odyssey, LI-COR Biosciences).

### 2.4. RNA Extraction and Real-Time PCR

Total RNA was extracted from mice liver using RNeasy Protect Mini kit (QIAGEN, Maryland, USA) according to the manufacturer's instructions. Total RNA (1 *μ*g) was transcribed into cDNA using GeneAmp kit (Applied Biosystems, Branchburg, NJ, USA) reverse transcription reagents. Total RNA was analyzed by a quantitative real-time polymerase chain reaction (qRT-PCR). Real-time PCR was performed using SYBR Green PCR Master Mix (Applied Biosystems) on a 7500 HT Fast Real-Time PCR System (Applied Biosystems). Specific primers for mouse ferritin heavy chain and hepcidin were used. The mouse ferritin heavy chain amplification primers were Fwd 5′-XXX-3′ and Rev 5′-XXX-3′, while the mouse hepcidin amplification primers were Fwd 5′-CCAGCCTGAGCAGCACCACC-3′ and Rev 5′-TTGAGGGGCTGCAGGGGTGT-3′ (Integrated DNA Technologies). Each reaction was performed in triplicate. The comparative threshold cycle (Ct) method was used to calculate the fold amplification as specified by the manufacturer. All experimental samples were normalized using 18s as an internal control.

### 2.5. Statistical Analyses

Statistical significance between experimental groups was determined by ANOVA with Tukey-Kramer post hoc analysis. The data are presented as means ± SE and the null hypothesis was rejected at *p* < 0.05, *n* ≥ 3.

## 3. Results

### 3.1. Phenotype of Lean and Obese Mice, in the Absence or Presence of CoPP

**Figure 1:**
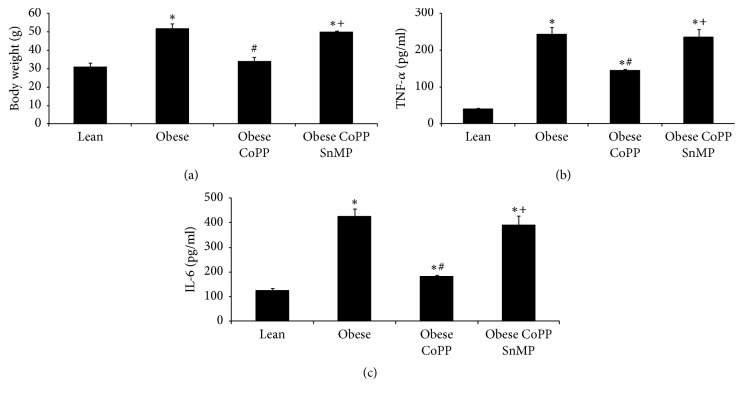
Effect of HO-1 induction on body weight and plasma levels of inflammatory cytokines in lean and obese mice. (a) Effect of CoPP and SnMP coadministration on body weight of obese mice after 6 weeks of treatment. The data are the weights in grams as mean ± SEM (*p* < 0.05). (b) TNF-*α* plasma levels and (c) IL-6 plasma levels results are mean ± SEM *n* = 3-4, ^*∗*^*p* < 0.05 versus lean; ^#^*p* < 0.05 versus obese; ^+^*p* < 0.05 versus obese CoPP.

As shown in Figure 1(a), obese mice weighed significantly (*p* < 0.05) more than lean mice. CoPP treatment prevented (*p* < 0.05) weight gain in obese mice and normalized body weight as compared with age-matched lean controls. This effect of CoPP was offset (*p* < 0.05) in obese mice concurrently treated with SnMP. Complementary experiments examining markers of chronic inflammation revealed significantly higher levels of TNF-*α* (*p* < 0.05) and IL-6 in obese mice, as compared to age-matched lean controls (Figures 1(b) and 1(c), resp.). Treatment of obese mice with CoPP resulted in a significant (*p* < 0.05) decrease in these proinflammatory cytokines; an effect that was prevented (*p* < 0.05) by SnMP treatment (Figures 1(b) and 1(c)).

Complementary experiments reveal elevated plasma glucose level in obese mice, which is prevented in CoPP treated mice. This reversal in plasma glucose is not seen in obese mice concurrently exposed to SnMP (Figures 1(a), 1(b), and 1(c)).

### 3.2. Redox Balance in Hepatic Tissues of Lean and Obese Mice, in the Absence or Presence of CoPP

**Figure 2:**
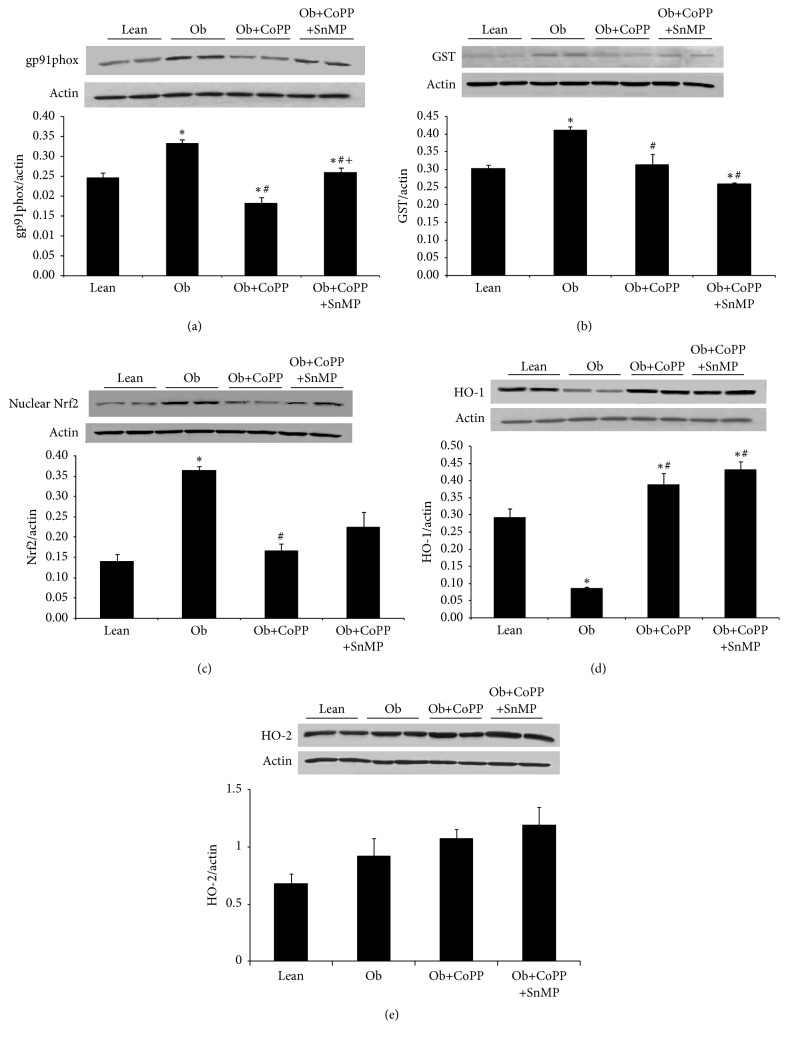
Effect of HO-1 induction on gp91phox, GST, and nuclear NRF2 protein levels in liver of lean and obese mice. Western blot and densitometry analysis of (a) gp91phox, (b) GST, (c) nuclear NRF2, (d) HO-1, and (e) HO-2 protein levels. Results are mean ± SEM of the band density normalized to actin. *n* = 3-4, ^**∗**^*p* < 0.05 versus lean; ^#^*p* < 0.05 versus obese; ^+^*p* < 0.05 versus obese CoPP.

The redox state of these mice was assessed in hepatic tissue via expression analysis of the oxidant enzyme, gp91phox, and by levels of antioxidant response proteins, that is, glutathione s peroxidase (GST) and Nrf2 (Figures 2(a), 2(b), and 2(c), resp.). Obese mice had significantly higher levels of oxidative stress as characterized by increased (*p* < 0.05) levels of gp91phox and the antioxidant proteins GST and Nrf2 (Figures 2(a)–2(c)). CoPP treatment (*p* < 0.05) increased gp91phox, GST, and Nrf2 levels, which is indicative of a restoration of the redox balance in obese mice (Figures 2(a)–2(c)).

In spite of increased nuclear Nrf2, a transcriptional coregulator of HO-1, HO-1 levels was not enhanced in obese mice; on the contrary, there was a significant (*p* < 0.05) attenuation of HO-1 (Figure 2(d)). HO-1 expression was enhanced (*p* < 0.05) in both CoPP and SnMP treated mice, without any effect on hepatic HO-2 levels (Figure 2(e)).

### 3.3. Iron Homeostasis in Lean and Obese Mice, Treated with and without CoPP

**Figure 3:**
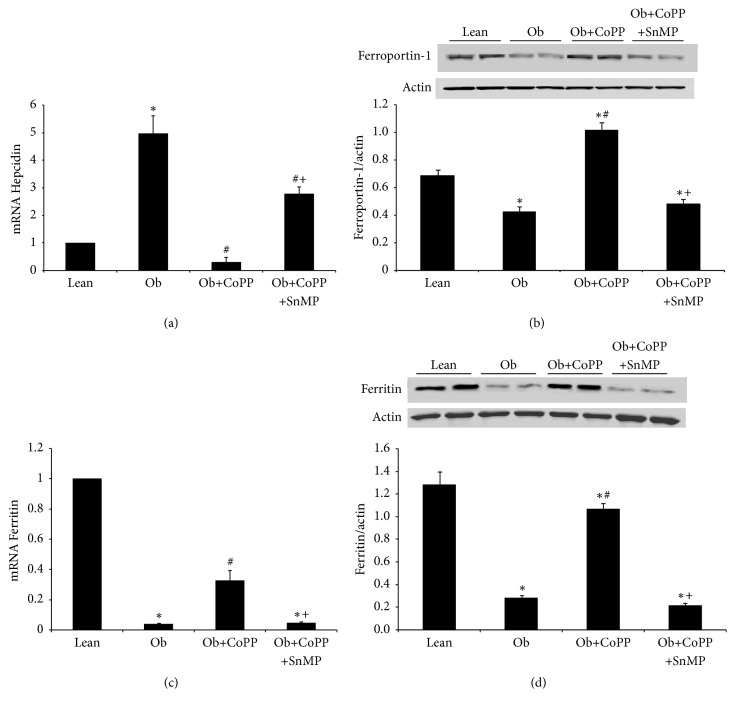
Effect of HO-1 induction on hepcidin, ferroportin, and ferritin levels in liver of lean and obese mice. (a) Hepcidin mRNA levels, (b) ferroportin protein levels, and ferritin mRNA and protein levels in (c) and (d), respectively. Results are mean ± SEM. *n* = 3-4, ^*∗*^*p* < 0.05 versus lean; ^#^*p* < 0.05 versus obese; ^+^*p* < 0.05 versus obese CoPP.

As shown in Figure 3(a), hepatic hepcidin expression was significantly (*p* < 0.05) increased in obese mice as compared to their lean counterparts. This phenotypic alteration was reversed (*p* < 0.05) in mice undergoing treatment with CoPP. Increased hepatic hepcidin affected cellular ferroportin levels as expected, demonstrating a significant decline in obese mice (Figure 3(b)). Reduced ferroportin facilitates cellular iron overload, thus activating translation of iron responsive genes, including ferritin. However, counterintuitively, hepatic ferritin levels were significantly lower in obese mice (*p* < 0.05) (Figures 3(c) and 3(d)). CoPP treatment rescued (*p* < 0.05) hepatic ferroportin and ferritin levels in obese mice. Cotreatment with SnMP, in CoPP treated obese mice, prevented the protective effects of CoPP on hepcidin, ferroportin, and ferritin expression, corroborating the role of heme-HO system in mediating these effects (Figures 3(a)–3(d)).

### 3.4. Effect of HO-1 Induction on Insulin Receptor in Liver Microsomal Fraction

**Figure 4:**
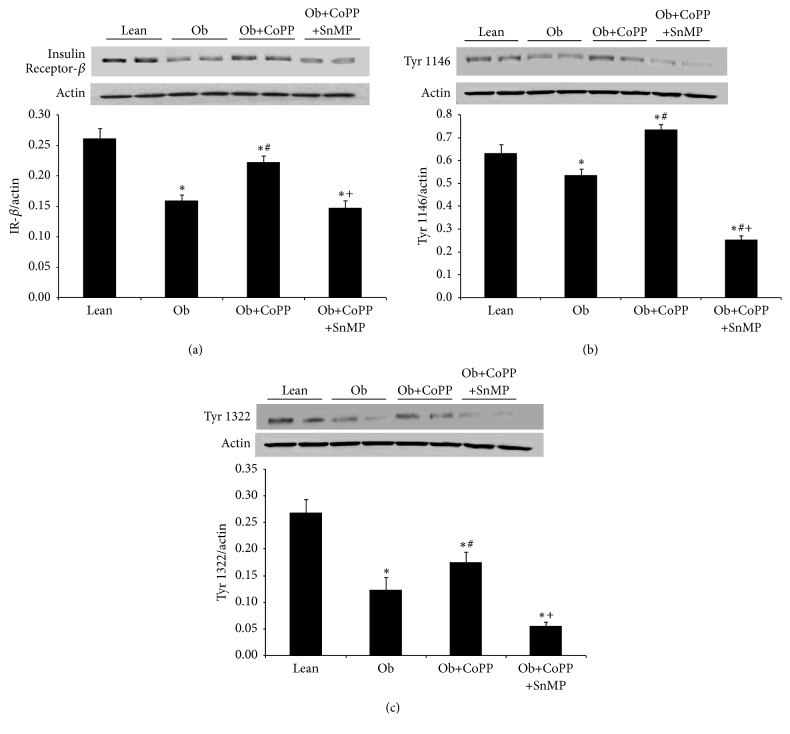
Effect of CoPP and SnMP administration on insulin receptor-*β*, insulin receptor phosphorylated at tyrosine 1146, and insulin receptor phosphorylated at tyrosine 1322 protein levels in liver of lean and obese mice. (a) Total insulin receptor-*β* (IR-*β*) protein levels, IR-*β* phosphorylation at (b) tyrosine 1146, and (c) tyrosine 1322. Results are mean ± SEM of the bands density normalized to actin. *n* = 3-4, ^**∗**^*p* < 0.05 versus lean; ^#^*p* < 0.05 versus obese; ^+^*p* < 0.05 versus obese CoPP.

Western blot analyses of generic insulin receptor beta showed a significant (*p* < 0.05) decrease in obese mice as compared with their lean controls (Figure 4(a)). This decrease was blocked by the administration of CoPP, while coadministration of CoPP and SnMP returned their levels to the untreated obese expression (Figure 4(a)). Similar results were observed in the expression of liver insulin receptor phosphorylated at tyrosine 1466 and at tyrosine 1322 (Figures 4(b) and 4(c)).

### 3.5. Effect of CoPP Treatment on pAMPK/AMPK and pAKT/AKT Ratio in Liver Microsomal Fraction

**Figure 5:**
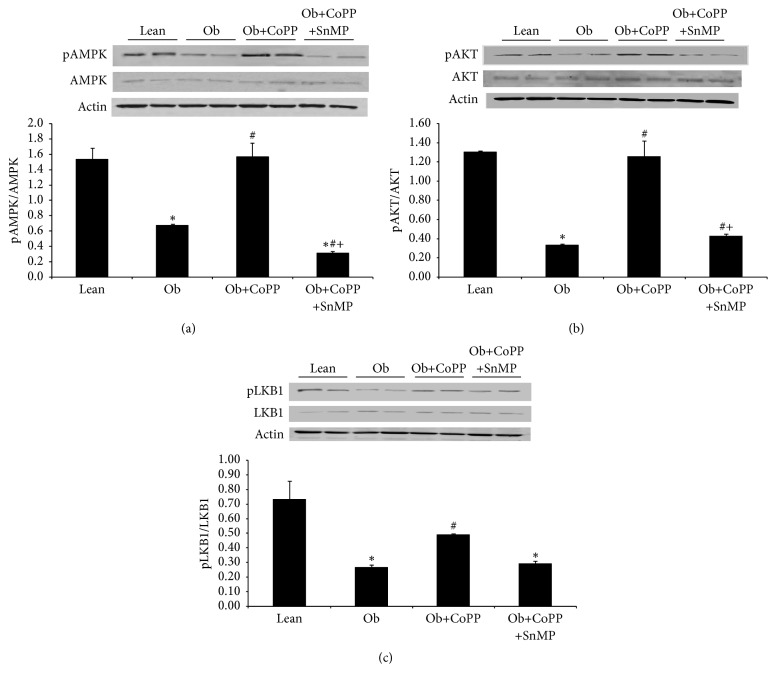
Effect of CoPP and SnMP administration on pAMPK/AMPK and pAKT/AKT protein levels in liver of lean and obese mice. (a) Total and phosphorylated AMPK, and pAMPK/AMPK, (b) total and phosphorylated AKT, and pAKT/AKT, and (c) total and phosphorylated LKB1, and pLKB1/LKB1. Results are mean ± SEM, *n* = 3-4, ^**∗**^*p* < 0.05 versus lean; ^#^*p* < 0.05 versus obese; ^+^*p* < 0.05 versus obese CoPP.

Downregulation of insulin receptors and their phosphorylation was complemented by reduced (*p* < 0.05) pAMPK/AMPK ratio in obese mice (Figure 5(a)). This decrease was blocked by the administration of CoPP, while the coadministration of CoPP and SnMP returned this ratio to the levels seen in untreated obese mice (Figure 5(a)). A similar pattern was observed in hepatic expression of pAKT and its upstream regulator, that is, pLKB1 (Figures 5(b) and 5(c)). As obese mice had suppressed (*p* < 0.05) levels of these metabolic regulators, CoPP treatment abated this effect (Figures 5(b) and 5(c)). The underlying role of HO-1 induction, via CoPP, in mediating these effects was corroborated by loss (*p* < 0.05) of the protective effects of CoPP in animals treated with the HO-1 inhibitor, SnMP (Figures 5(b) and 5(c)).

### 3.6. Effect of CoPP Treatment on Mitochondria Energy Expenditure- and Fusion-Associated Proteins

**Figure 6:**
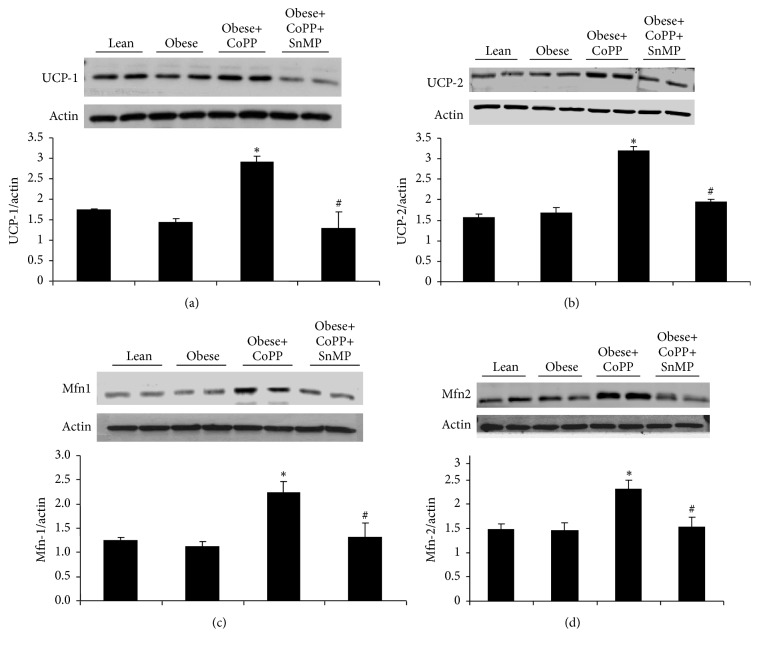
Effect of CoPP and SnMP administration on UCP-1 and UCP-2, and Mfn-1 and Mfn-2 protein levels in liver of lean and obese mice. Representative blots and associated graphs depicting densitometric analysis relative to *β*-actin of (a) UCP-1, (b) UCP-2, (c) Mfn-1, and (d) Mfn-2. Results are mean ± SEM, *n* = 3-4, ^**∗**^*p* < 0.05 versus obese control; ^#^*p* < 0.05 versus obese treated with CoPP.

Western blot analyses of uncoupling proteins 1 and 2 showed a significant (*p* < 0.05) increase in obese mice treated with CoPP as compared with their obese controls (Figures 6(a) and 6(b)). This increase was blocked by the concomitant administration of SnMP (Figures 6(a) and 6(b)). Similar results were observed in the expression of mitochondrial fusion-associated proteins mitofusins 1 and 2 (Figures 6(c) and 6(d), resp.).

## 4. Discussion

*The first key finding* presented in this study is the suppressive effect of HO-1 induction on hepatic hepcidin levels in a murine model of obesity. Hepcidin is secreted mostly from hepatocytes and less from macrophages and plays a central role in iron homeostasis [28]. An elevated hepcidin level inhibits duodenal iron absorption and is implicated as the central pathophysiological alteration in anemia of chronic disease [6, 29]. Plasma hepcidin levels are increased in chronic inflammatory states frequently associated with oxidative stress, such as obesity, metabolic syndrome, and atherosclerosis [6, 30–32]. It is noteworthy, however, that some studies have also shown reduced hepcidin levels in conditions of oxidative stress [33]. Regardless, higher circulating levels of hepcidin can induce downregulation of the iron transport protein ferroportin from cellular plasma membranes throughout the body [7]. We show here that obese mice have increased hepatic hepcidin expression with a concomitant suppressive effect on ferroportin, the sole egress portal for excess cellular iron. These pathological alterations, together, have the potential to promote intracellular iron accumulation. Excess free iron is not only cytotoxic but also promotes oxidative stress via facilitating conversion of hydrogen peroxide to the hydroxyl radical (OH^•^), the Fenton reaction. The hydroxyl radical is one of the most reactive free radicals in biological systems and reacts near its diffusion limit. It can cause membrane damage, DNA and RNA fragmentation, and lipid peroxidation, while contributing to redox imbalance [34].

In an iron-replete cell, excess iron is exported from the cell via a transmembrane protein, ferroportin. In chronic inflammatory states, such as obesity, hepcidin-induced cytoplasmic recycling and lysosomal degradation of ferroportin trap excess iron in the cell [5–7]. This free iron forms a [4Fe-4S] cluster with iron regulatory protein 1 (IRP1), thus disengaging it from iron response elements (IRE) [35]. IRP1 controls the translation, and/or stability, of several mRNAs of iron responsive genes by binding to iron responsive elements within their untranslated regions. Ferritin has the capacity to bind thousands of ferric ions, thus keeping them out of solution. Intracellular iron overload, a consequence of ferroportin downregulation, posttranscriptionally activates ferritin expression by delivering it from the inhibitory effect of IRP1 [36]. However, the prooxidant properties of iron predispose the iron overloaded cell to redox imbalance. This iron-induced oxidative stress can destabilize the cubane [4Fe-4S] clusters [37].

Ferritin upregulation, in parallel to iron accumulation, is central to the sustenance of iron homeostasis. Apart from keeping iron out of solution in the cytosol, ferritin also has antioxidant [38] and anti-inflammatory properties [39].

*The second key finding* presented in this study delineates the facilitatory role of HO-1 in cellular ferritin levels. Rescue of ferritin expression, in CoPP treated mice, highlights the role of HO-1-mediated antioxidant effect in allowing ferritin upregulation in face of a cellular iron overload. Antioxidant properties of HO-1 have been largely ascribed to generation of BV, which reduces oxidative stress via attenuation of lipid peroxidation. BV-dependent restoration of redox homeostasis could offset the inhibitory effect of ROS on IRP-[4Fe-4S] complex, thus, allowing disengagement of IRP-1 from and translation of the ferritin mRNA. Consequentially, this circuitous loop of iron-induced oxidative stress suppressing ferritin, leading to increased free iron, which in turn enhances ROS generation, is interrupted by HO-1 upregulation.

Another possibility for reduced ferritin expression could be the inflammatory milieu of obesity. It is plausible that inflammatory cytokines, by a yet unknown mechanism, may be disrupting ferritin regulation. It could also be interplay of an oxidative redox state and the inflammatory milieu. In any case, HO-induced rescue of ferritin is quite remarkable and could be linked to either the antioxidant or the anti-inflammatory or an unrelated effect of HO. Although CoPP may have affected other signaling pathways in cells, our conclusions of HO-mediated ferritin rescue are strengthened by the fact the HO-inhibitor is able to block ferritin rescue in obese mice treated with CoPP. Another potential limitation of these conclusions is the fact that we did not measure plasma ferritin or transferrin levels. It is unlikely but possible that cellular levels are different from those in the plasma.

Enhanced ROS generation contributes to insulin resistance and dysregulation of downstream metabolic targets, that is, AMPK, LKB1, and AKT. P-AMPK is known to act in the regulation of cell survival, protect against oxidative stress, and, when activated, contribute to glucose transport, fatty acid oxidation, and increased mitochondrial function. LKB1 is a serine-threonine kinase that directly phosphorylates AMPK and improves glucose tolerance. Hepatic AMPK activation facilitates [40] insulin-dependent inhibition of gluconeogenesis. Redox-dependent attenuation of LKB1 phosphorylation [41] prevented AMPK activation in obese mice. Lack of AMPK activation increases hepatic glucose output and contributes to hyperglycemia, while furthering insulin resistance.

*Our third key finding demonstrates* that HO-1 induction, in obese mice, lowers cellular redox and enhances AMPK and AKT phosphorylation. These changes are accompanied by an improved metabolic profile in these animals exemplified by reduced body weight and lowering of blood glucose levels. Also, HO-1/HO-2 are involved in regulation of mitochondrial biogenesis, quality control, function, and levels of cytochrome oxidase as well as apoptosis [4, 42–45] and we have shown that HO-1 is present in the mitochondrial inner membrane and cortex [42]. We found that CoPP-mediated induction of HO-1 in obese mice showed improved mitochondrial energy expenditure and increased fusion-associated parameters as compared to obese control mice. Even though a HF diet itself did not reduce Mfn and UCP levels in liver tissues, the CoPP-mediated increase was effectively reduced in mice concomitantly treated with the HO-inhibitor, SnMP, corroborating our recent report showing that impaired HO-expression and activity decrease mitochondrial function in adipose tissues of obese mice and 3T3-L1-derived adipocytes, contributing to increased mitochondrial derived superoxide formation [12, 46]. Mice deficient in Mfn1, Mfn2, and OPA1 are all embryonic lethal [47] and in humans, mutations in OPA1 are linked to hereditary blindness, and Mfn2 mutations are known to cause Charcot-Marie-Tooth disease [47–49]. In mice, a liver-specific knockout of Mfn2 is associated with impaired insulin signaling and glucose metabolism [50]. Our results show that HO-1 induction increases mitochondrial energy metabolism and fusion-associated processes are supported by a recent report in which doxorubicin-induced oxidative stress increased mitochondrial fragmentation and Fis1 expression, an effect that was prevented by HO-1 induction favoring expression of Mfn1 and Mfn2 [18, 45]. It should be noted, however, that antioxidant and anti-inflammatory properties of the HO may not be observed under all conditions [51]. In contrast, humans with deleted HO-1 suffer severe oxidative stress and organ failure [13, 14, 52, 53]. These authors further showed that a defect in iron recycling accounts for dysregulation of iron homeostasis in human with heme oxygenase-1 deficiency [17].

This does not appear to translate to the presentation of NAFLD in humans. Two-thirds of the US population are overweight or obese [54]. 30% of the US population has NAFLD. Serum ferritin is an independent predictor of advanced fibrosis and even histologic severity in NAFLD [55, 56]. Iron metabolism appears to be altered in NAFLD. Most food contains glucose and iron, resulting in hyperglycemia, diabetes, and iron overload [57]. This iron overload stimulates hepcidin secretion. It is not clear whether the elevated hepcidin secretion is from the inflammation of obesity or the inflammatory effects of iron overload, or both. Only 20% of these patients have elevated ferritin levels, which is associated with advanced liver fibrosis [55].

It is clear that the results of our study show that iron metabolism is altered in NAFLD. What is unclear is how ferritin goes from having a protective role in a normal liver to being the independent predictor of advanced fibrosis. More work needs to be done to unravel the dilemma of iron metabolism alterations in NAFLD.

Thus, our results show that chronic low-grade inflammation and oxidative stress promote hepatic hepcidin secretion which then attenuates cellular ferroportin levels. These events could create an iron overload in the cell, whose prooxidant properties exacerbate the metabolic imbalance and interfere with upregulation of ferritin. HO-1 induction attenuates hepatic hepcidin synthesis and restores redox balance. Consequently, rescue of ferroportin and ferritin expression counteract cellular iron overload and abate iron-induced oxidative stress. The pathophysiological impact of HO-1 induction includes improved mitochondrial energetics and overall improvement of the mouse-metabolic profile. Future studies in mouse-models of nonalcoholic fatty liver and nonalcoholic steatohepatitis may provide additional evidence to support the role of HO-induction in the treatment of these metabolic disorders.
